# Oral HIV pre-exposure prophylaxis use and resistance-associated mutations among men who have sex with men and transgender persons newly diagnosed with HIV in the Netherlands: results from the ATHENA cohort, 2018 to 2022

**DOI:** 10.2807/1560-7917.ES.2024.29.38.2400083

**Published:** 2024-09-19

**Authors:** Vita W Jongen, Daniela Bezemer, Ard van Sighem, Anders Boyd, Casper Rokx, Karin Grintjes, Aafke Cents-Bosma, Eline Op de Coul, Birgit van Benthem, Annemarie Wensing, Ferdinand WNM Wit, Marc van der Valk, on behalf of the ATHENA national observational HIV cohort

**Affiliations:** 1 Stichting HIV Monitoring, Amsterdam, the Netherlands; 2 Department of Infectious Diseases, Public Health Service Amsterdam, Amsterdam, the Netherlands; 3 Amsterdam University Medical Centers, University of Amsterdam, Department of Infectious Diseases, Amsterdam Infection & Immunity Institute, Amsterdam, the Netherlands; 4 Department of Internal Medicine, Section Infectious Diseases, and Department of Medical Microbiology and Infectious Diseases, Erasmus University Medical Center, Rotterdam, the Netherlands; 5 Department of Internal Medicine, Radboud University Medical Center, Nijmegen, the Netherlands; 6 Flevoziekenhuis, Almere, the Netherlands; 7 Centre for Infectious Disease Control, National Institute for Public Health and the Environment (RIVM), Bilthoven, the Netherlands; 8 Translational Virology Research Group, Department of Medical Microbiology, University Medical Center, Utrecht, the Netherlands; 9 The members of ATHENA national observational HIV cohort are acknowledged at the end of the article

**Keywords:** Pre-exposure prophylaxis, HIV prevention & control, Men who have sex with men

## Abstract

**Background:**

In the Netherlands, HIV pre-exposure prophylaxis (PrEP) has been available since 2019. However, the extent of PrEP use prior to HIV diagnosis and development of PrEP-resistance-associated mutations (RAMs) is not known.

**Aim:**

We assessed prior PrEP use and potential transmission of PrEP RAMs among men who have sex with men (MSM) and transgender persons (TGP) with a new HIV diagnosis in the Netherlands.

**Methods:**

Data on prior PrEP use between 1 January 2018 and 31 December 2022 were available from the Dutch national ATHENA cohort. We assessed proportion of prior PrEP use, detected PrEP associated RAMs and assessed potential onward transmission of RAMs between 2010 and 2022 using a maximum likelihood tree.

**Results:**

Data on prior PrEP use were available for 583/1,552 (36.3%) individuals, with 16% (94/583) reporting prior PrEP use. In 489 individuals reporting no prior PrEP use, 51.5% did not use PrEP due to: low HIV-risk perception (29%), no access (19.1%), personal preference (13.1%), and being unaware of PrEP (19.1%). For PrEP users, 13/94 (13.8%) harboured a M184V/I mutation, of whom two also harboured a K65R mutation. In people with a recent HIV infection, detection of PrEP RAMs increased from 0.23% (2/862) before 2019 to 4.11% (9/219) from 2019. We found no evidence of onward transmission of PrEP RAMs.

**Conclusion:**

The prevalence of PrEP-associated RAMs has increased since PrEP became available in the Netherlands. More widespread access to PrEP and retaining people in PrEP programmes when still at substantial risk is crucial to preventing new HIV infections.

**Key public health message**

**What did you want to address in this study and why?**

Suboptimal adherence to oral pre-exposure prophylaxis (PrEP), combined with exposure to HIV, can result in HIV infection and selection of resistance-associated mutations. This could limit effectiveness of PrEP in the population at risk and treatment options for individuals with HIV. We assessed prior PrEP use and prevalence of PrEP-associated mutations in a national database of HIV infections in men who have sex with men and transgender persons.

**What have we learnt from this study?**

Only 16% of individuals with a new HIV diagnosis reported using PrEP prior to their diagnosis. Low perceived risk of HIV, no access to PrEP and being unaware of PrEP were reasons for not using PrEP. The prevalence of PrEP-associated mutations increased after 2019 – the year in which PrEP use was widespread in the Netherlands – but we found no evidence for onward transmission of these mutations.

**What are the implications of your findings for public health?**

Some individuals are still acquiring HIV despite previous or current PrEP use. Moreover, although there is an increase in the prevalence of PrEP-associated resistance mutations, the lack of any clear transmission patterns of these mutations suggests, transmission of these mutations is rare. An improved understanding of the risk of HIV is needed for certain PrEP users, and frequent HIV testing is crucial.

## Introduction

Oral pre-exposure prophylaxis (PrEP), when taken correctly, is highly effective in preventing the acquisition of human immunodeficiency virus (HIV) [1,2]. However, adequate adherence is key to assuring effective protection [1]. Exposure to HIV in combination with poor adherence can result in the acquisition of HIV and selection of resistance-associated mutations to emtricitabine (FTC) and/or tenofovir (TDF), the two active agents used in oral PrEP [3]. If onward transmission of these strains occurs, the effectiveness of PrEP in the population at risk as well as the effectiveness of antiretroviral treatment (ART) in people living with HIV could theoretically decrease [4]. To date, failure of PrEP due to the acquisition of a PrEP-resistant virus has been sporadically reported [3,5-8]. In some of these cases, HIV seroconversion may be delayed, posing challenges to HIV diagnosis [9].

In the Netherlands, oral PrEP (co-formulated as TDF/FTC) was first available through prescription by individual healthcare practitioners, demonstration projects or clinical trials [10] and informal means. Since August 2019, PrEP has been available through a National PrEP programme, offering PrEP as either a daily or event-driven regimen. As of April 2024, the programme has a limited capacity of 8,500 people at high risk of acquiring HIV, which mainly includes men who have sex with men (MSM) and transgender persons. Part of the programme allows PrEP users to be screened for HIV and sexually transmitted infections every 3 months. The demand for PrEP through the PrEP programme has far exceeded capacity and those wanting to be included have been wait-listed and are required to pay out-of-pocket for PrEP from other sources e.g. general practitioner, other commercial health practice. From August 2024, the budget for PrEP care in the Netherlands has increased, but PrEP is longer partially reimbursed and PrEP users have to pay out-of-pocket for their PrEP.

To gain more insight into the involvement of PrEP use on newly diagnosed HIV infections, we used data from the Dutch National AIDS Therapy Evaluation in the Netherlands study (ATHENA cohort) to assess prior PrEP use from 2018 onwards among those newly diagnosed, and the occurrence and transmission of PrEP-resistant HIV-1 strains over time coinciding with expanded access to PrEP on a national level.

## Methods

### Study design

In the Netherlands, HIV care is provided by 24 designated treatment centres. The Dutch HIV Monitoring Foundation (SHM) is tasked by the Ministry of Health, Welfare and Sport to continuously monitor and report on all aspects of HIV in the population of people with HIV. Since 1998, data have been collected by the ATHENA cohort from over 98% of all people with HIV who are receiving care in the Netherlands [11].

For this analysis, we included data from MSM and transgender persons newly registered with HIV in the ATHENA cohort between 1 January 2010 and 31 December 2022. We did not include individuals born outside the Netherlands with a documented pre-migration HIV diagnosis.

### Study procedure

Demographic data and relevant HIV and treatment data are prospectively and continuously collected in the cohort. Information about the date of HIV diagnosis was retrieved from the referral letter of the general practitioner or Centre of Sexual Health, from health records at the HIV treatment centre, or self-reported if no documentation was available. Data relating to PrEP (i.e. prior PrEP use and reasons for not using PrEP) and genotypic resistance test results were collected after entering into HIV care from electronic medical records from January 2018 onwards. Data on PrEP were collected in collaboration with the Dutch Association of HIV-Treating Physicians and the Dutch Association of HIV nurse consultants.

HIV-1 partial polymerase gene sequences are obtained as part of routine screening for antiretroviral drug resistance [12]. Resistance-associated mutations (RAMs) to FTC or TDF at positions K65R/E/N, K70E, and M184V/I (which is also associated with resistance to lamivudine) of the RT gene were recorded and analysed for transmission if a sequence was available in the database of the HIV Monitoring Foundation [13].

### Phylogenetic analysis

To investigate whether there is any evidence of onward transmission of strains with PrEP-related RAMs, we applied two methods, one under loose criteria selecting subtrees from a phylogenetic tree, and the other based on a distance cut-off to select clusters of closely-related strains independent of a phylogenetic tree. The first available sequence from MSM and transgender persons diagnosed with HIV between 2010 and 2022 with a polymerase sequence available before starting antiretroviral treatment, and a minimal length of 600 nt of the reverse transcriptase (RT) gene, was included in this analysis. The resulting sequence alignment was cut at 1,200 nucleotides. A phylogenetic tree was built using a general time-reversible model in FastTree (version 2.1.3) [14,15]. Phylopart 2.0 [16] was used to obtain subtrees with a local support value of ≥ 0.9 based on 1,000 resamples, and a median value of all pairwise patristic distances of the subtree below the 1st percentile threshold for the distribution of all pairwise distances of the entire tree (corresponding to 0.08 mutations per site). HIV-TRACE was used to identify clusters of closely related strains as sequences with ≤ 1.5% genetic divergence [17]. Transmission of resistant strains were investigated on subtrees and clusters of MSM and transgender persons who had PrEP-associated RAMs at time of HIV diagnosis.

### Statistical analysis

The annual number of new and recent HIV diagnoses between 2010 and 2022 were expressed as absolute numbers. Recent HIV infections were defined as those determined by a negative or indeterminate HIV Western blot at the time of diagnosis (confirmed with a positive serum HIV-RNA PCR test) or a negative HIV test ≤ 12 months before diagnosis. Among individuals with a new HIV diagnosis between 2018 and 2022, we calculated the proportion of individuals who reported prior PrEP use over time. We also calculated the proportion with HIV strains harbouring major RAMs to TDF or FTC and treatment response to ART in the overall population with a new HIV diagnosis and in those with a recent HIV infection.

All analyses were performed using SAS statistical software version 9.4 (SAS, Cary, United States).

## Results

**Figure 1:**
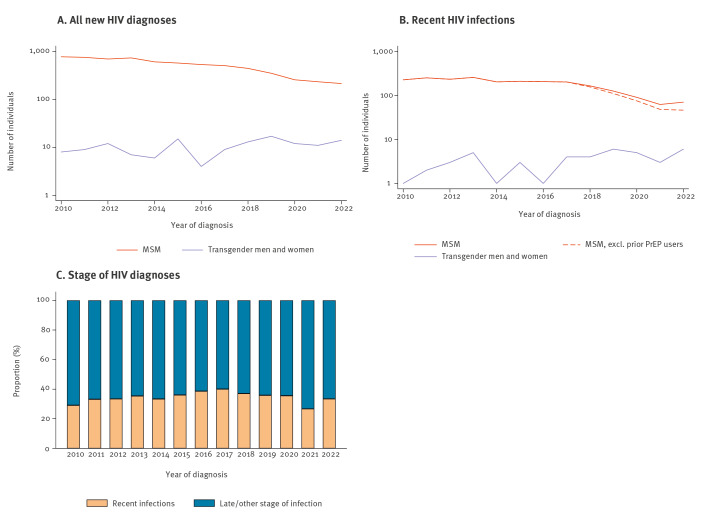
(A) Total number of new HIV-1 diagnoses, (B) recent HIV infections^a^ and (C) HIV diagnosis stage for men who have sex with men and transgender persons, in the ATHENA cohort, the Netherlands, 2010–2022

Between 2010 and 2022, there were 6,623 new HIV diagnoses among MSM and 137 among transgender persons. Among MSM, the annual number of new HIV diagnoses decreased by 72%, from 768 in 2010 to 213 in 2022 (Figure 1A). Among transgender persons, the annual number of new HIV diagnoses fluctuated between 4 and 17 during the same period (Figure 1A). The proportion of MSM and transgender persons with evidence of a recent HIV infection increased until 2018, and then decreased (Figure 1B and 1C).

ATHENA cohort: Dutch National AIDS Therapy Evaluation in the Netherlands study; MSM: men who have sex with men; PrEP: pre-exposure prophylaxis.

^a^ A recent HIV infection was defined as a negative or indeterminate HIV Western blot at the time of diagnosis or a last negative HIV test ≤ 12 months before diagnosis.

The number of individuals in (A) and (B) is shown on the log_10_ scale.

### Prior pre-exposure prophylaxis use

**Figure 2:**
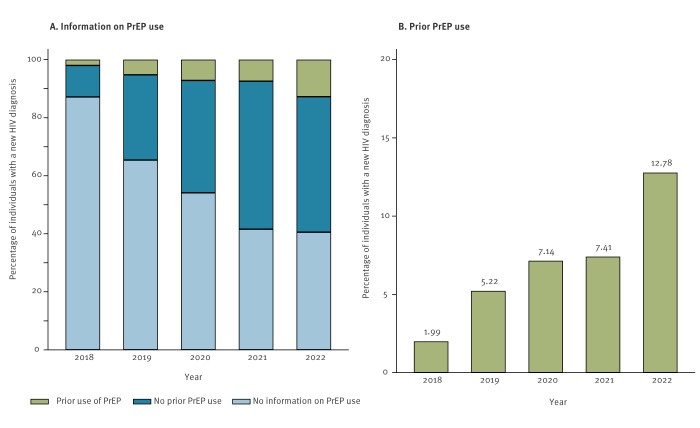
(A) Information available on oral pre-exposure prophylaxis use and (B) prior oral pre-exposure prophylaxis use in men who have sex with men and transgender persons with a new HIV diagnosis, ATHENA cohort, the Netherlands, 2018–2022

Data on prior PrEP use were available for 583 (36.3%) of 1,552 MSM and transgender persons newly diagnosed with HIV between 1 January 2018 and 31 December 2022 (Figure 2A).

ATHENA cohort: Dutch National AIDS Therapy Evaluation in the Netherlands study; PrEP: pre-exposure prophylaxis.

Socio-demographic characteristics of individuals with and without information on PrEP were similar (Table 1). Individuals with information on PrEP use were more often diagnosed with a recent HIV infection and had a higher CD4^+^ T-cell count (median: 435 cells/mm^3^, interquartile range (IQR): 251–619) at the time of diagnosis than individuals without information on PrEP (median: 390 cells/mm^3^, IQR: 201–580). Of the 583 individuals with information on prior PrEP use, 16.1% reported PrEP use before being diagnosed with HIV (93 MSM and one transgender person). Annually, the proportion of newly diagnosed MSM and transgender persons reporting prior PrEP use has increased since 2019 after the national PrEP programme was initiated (Figure 2B). Twenty-six individuals (28%, 26/94) reported that they had still been using PrEP at the time of HIV diagnosis. Median time between last PrEP dose and HIV diagnosis was 77 days (IQR: 0–224). For individuals who discontinued PrEP before HIV diagnosis, median time between last reported PrEP intake and HIV diagnosis was 121 days (IQR: 60–231).

**Table 1:** Characteristics of individuals with a new HIV diagnosis and information on PrEP use, the Netherlands, 2018–2022

Characteristics	Prior PrEP use (n = 94)		No PrEP use (n = 489)		No information on PrEP use available (n = 969)	
	n^a^	%^a^	n^a^	%^a^	n^a^	%^a^
**Median age (IQR)**	32.0 (26.9–41.1)		37.8 (29.5–50.0)		39.2 (28.9–49.9)	
**Gender**						
Cisgender MSM	93	98.9	462	94.5	930	96.0
Transgender male	1	1.1	3	0.6	3	0.3
Transgender female	0	0	24	4.9	36	3.7
**Born in the Netherlands**						
No	49	52.1	291	59.5	390	40.2
Yes	45	47.9	198	40.5	579	59.8
**Recent HIV acquisition^b^**						
Tested pos < 365 days after last neg test	73	77.7	145	29.7	240	24.8
Tested pos < 180 days after last neg test	48	51.1	90	18.4	117	12.1
**Viral load and CD4^+^ T-cell at HIV diagnosis**						
CD4^+^ T-cell count at HIV diagnosis (cells/mm^3^), median (IQR)	540 (347–716)		408 (240–590)		390 (201–580)	
Viral load < 200 c/mL at HIV diagnosis	4/86	4.7	14/446	3.1	21/899	2.3
**Days to first viral load < 50 c/mL after start ART, median (IQR)^c^**	64 (30–116)		68 (30–121)		79 (33–132)	

ART: antiretroviral treatment; IQR: interquartile range; MSM: men who have sex with men; neg: negative; pos: positive; PrEP: pre-exposure prophylaxis.

^a^ Unless otherwise indicated.

^b^ A recent HIV infection was defined as a negative or indeterminate HIV Western blot at the time of diagnosis or a last negative HIV test ≤ 12 months before diagnosis.

^c^ Ninety of 94 individuals who indicated prior PrEP use achieved a viral load < 50 copies/mL after ART initiation.

Among the four individuals who had not yet achieved an undetectable viral load, one had not started ART at the time of writing, two did not have a viral load measurement after ART initiation and one had a latest measured viral load of 61 copies/mL.

For 252 of the 489 (51.5%) MSM and transgender persons who reported no prior PrEP use, information on reasons for not using PrEP was available: 72 (28.6%) presumed themselves to be at low risk for HIV, 48 (19.1%) wanted to use PrEP but were unable to get access, 33 (13.1%) knew about PrEP but preferred not to use it, and 48 (19.1%) reported being unaware of PrEP. Forty-seven (18.7%) individuals indicated that they had wanted to start PrEP before their HIV diagnosis but tested positive for HIV before entry into the PrEP programme. Four (1.6%) individuals reported that they seroconverted while on the waiting list for the PrEP programme.

### Pre-exposure prophylaxis-associated resistance mutations

Genotypic resistance test results at HIV diagnosis were available for 77 of the 94 (81.9%) MSM and transgender persons reporting prior PrEP use. Clinically relevant resistance mutations associated with PrEP use were detected in 13 (16.9%) of these 77 individuals (13.8% of all individuals with prior PrEP use). Eleven of these 13 were still using PrEP at HIV diagnosis and two had stopped shortly before HIV diagnosis. Of note, the exact timing of the HIV infection is unknown and PrEP use at the time of infection is unknown, but likely. In all 13 individuals, an M184V/I mutation was detected and in two of these individuals a K65R mutation was also detected (Table 2). All individuals either reported PrEP use at the time of HIV diagnosis or PrEP use several weeks before HIV diagnosis. Data on first-line ART and subsequent virological treatment response were available for all 13 individuals with PrEP-associated resistance mutations. All started a regimen containing an integrase inhibitor, which was combined with two nucleoside-analogue RT inhibitors (NRTIs, n = 5), or with a protease inhibitor with or without additional NRTIs (n = 8). Eleven of the 13 individuals with PrEP-associated mutations had a documented optimal treatment response (defined as HIV viral load < 50 copies/mL) after treatment initiation. One individual had a viral load between 50 and 100 copies/mL up until 16 months after ART initiation. Another individual with an M184V (but without K65R) resistance mutation at treatment initiation, switched to another triple-class regimen (consisting of two NRTI, an integrase strand transfer inhibitor (INSTI) and a boosted protease inhibitor) due to persistent low-level viraemia on their initial ART regimen (highest recorded value was 253 copies/mL). Viral load then became undetectable and remained undetectable for four consecutive measurements over a time span of over 1 year.

**Table 2:** Resistance-associated mutations to emtricitabine or tenofovir in the reverse transcriptase gene (K65R/E/N, K70E, and M184V/I) at diagnosis^a^ in men who have sex with men and transgender persons, the Netherlands, 2018–2022

**Person**	**Prior PrEP use**	**Year of diagnosis**	**Recent infection**	**RT mutations**	**Subtree^b^**	**Cluster**	**Subtype**
1	Yes	2018	Yes	184V	1287	7	B
2	Yes	2018	Yes	M184V	NA^c^	NA^c^	NA^c^
3	Yes	2019	No	184V,138A	4715	352	B
4	Yes	2020	Yes	184V	Singleton	Singleton	B
5	Yes	2020	Yes	184I	1949	135	F1
6	Yes	2020	Yes	184MI	4985	Singleton	B
7	Yes	2021	Unknown	184V	Singleton	Singleton	B
8	Yes	2021	Yes	65R,184V,108I,138A	6200	Singleton	B
9	Yes	2021	Yes	K65R, M184V	NA^c^	NA^c^	NA^c^
10	Yes	2022	Yes	184V	1190	Singleton	B
11	Yes	2022	Yes	184IMV	2022	6	F1
12	Yes	2022	Yes	184IMV	4844	371	B
13	Yes	2022	Yes	184MI	5730	9	B
14	Unknown	2011	Unknown	184IMV	3003	4	B
15	Unknown	2011	No	184VM	4601	Singleton	B
16	Unknown	2011	No	70EGKR,190A	5459	Singleton	B
17	Unknown	2013	No	70RK	3886	2	B
18	Unknown	2014	Yes	70E,138A	5335	Singleton	B
19	Unknown	2018	Yes	184V,103N,108I,225H	4543	Singleton	B
20	Unknown	2020	Yes	184I	2431	333	B

NA: not available; PrEP: pre-exposure prophylaxis; RT: reverse transcriptase.

^a^ Among all individuals for whom data on RT mutations were available, regardless of whether information on prior PrEP use was available.

^b^ In the phylogenetic tree in the Supplement, the leaf names show the subtree names. 0 indicates a singleton subtree.

^c^ For these patients, no sequence data were available for analysis. Only the results from the genotype resistance test were available.

### Transmission of pre-exposure prophylaxis-associated resistance mutations

**Figure 3:**
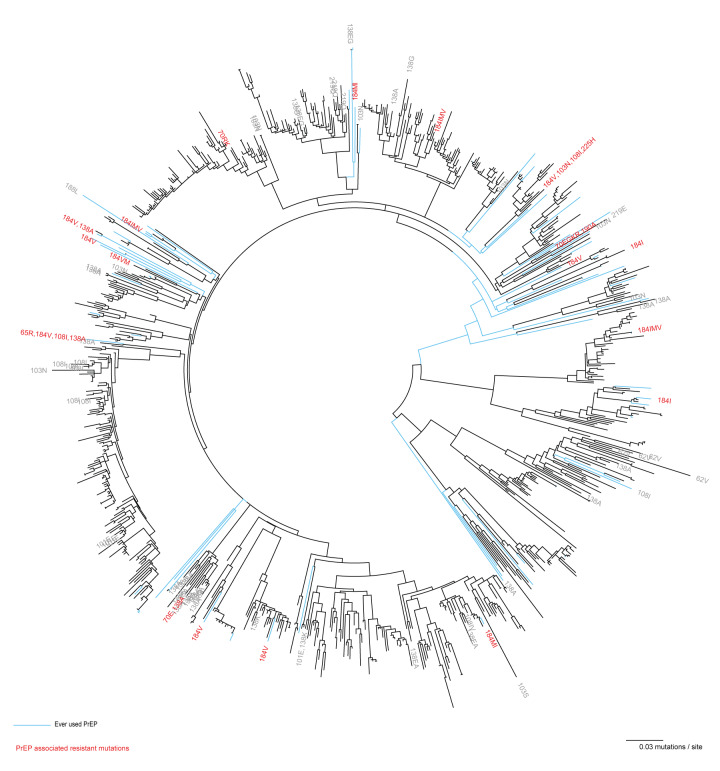
Maximum likelihood phylogenetic tree based on HIV-1 polymerase sequences selected from subtrees^a^ that contained sequences from individuals who had used PrEP or individuals diagnosed with PrEP-related resistance mutations, 2010–2022

A phylogenetic tree was built with sequences from 3,054 MSM and transgender persons. The full phylogenetic tree is available in the Supplement. Figure 3 is a phylogenetic tree of a selection of HIV-1 polymerase sequences in subtrees with either a sequence from individuals who had used PrEP, or individuals diagnosed with PrEP-related resistance mutations.

PrEP: pre-exposure prophylaxis.

^a^ Subtrees are described in the Supplement.

The leaf names show resistance-associated mutations in the reverse transcriptase gene [13]. PrEP-related leaf names are shown in red. Built with PhyML in Seaview [34] with a GTR model.

There were 65 sequences from individuals who indicated prior PrEP use - 64 MSM and one transgender person. These included 11 of the 13 individuals with PrEP RAMs reported above (Table 2). In addition, sequences were included from 2,989 MSM and transgender persons diagnosed in the Netherlands between 2010 and 2022 who did not report prior PrEP use, or for whom prior PrEP use was unknown. Among these, three MSM harboured HIV strains with mutations at position 70 of the RT gene (in 2011, 2013 and 2014). Two of the three also had strains harbouring a 70E mutation associated with low-level resistance to tenofovir), and four MSM had strains harbouring a mutation at RT184 (two in 2011, one in 2018, and one in 2020) (Table 2).

When inspecting the tree, there was no indication of onward transmission of strains with PrEP-related RAMs. All 18 sequences with PrEP-related RAMs from individuals before the start of ART were in different subtrees, 2 (11%) as singletons, and all 18 sequences were detected in different clusters, 9 (50%) as singletons (Table 2). Overall, in this sequence selection, the prevalence of RT M184V increased from four (0.16%) sequences from 2,488 individuals diagnosed with HIV before the initiation of the National PrEP programme (2010–2018) to 11 (1.96%) sequences from 566 individuals diagnosed since 2019. When the analysis was limited to individuals with evidence of a recent infection, and with a sequence obtained within 1 year after diagnosis and before the start of treatment, the prevalence of RT M184V increased from 0.23% before 2019 (the start of the national PrEP programme, 2/862) to 4.11% (9/219) in 2022.

## Discussion

We report that since the initiation of the national PrEP programme, at least 16% of MSM and transgender persons with a new HIV diagnosis used PrEP before being diagnosed. Moreover, the proportion of PrEP use among newly diagnosed MSM and transgender persons has risen rapidly since the initiation of the national PrEP programme. Clinically relevant PrEP RAMs were found in 13.8% of individuals who reported prior PrEP use. We also found that, using data from routinely collected surveillance-based sequencing among individuals with a recent HIV infection, the prevalence of RT M184V increased from 0.23% before to 4.11% after implementation of the national PrEP programme. Fortunately, this did not have any consequences on the response to first-line ART and we found no evidence of transmission of these resistant strains.

As PrEP is highly effective [1,2], making it widely accessible is crucial to decreasing the number of new HIV infections. Modelling studies have shown that PrEP could have a substantial impact on HIV incidence among MSM [18-20]. Empirical studies from Australia demonstrated an approximate 40% decrease in recent HIV infections up to 3 years after the start of their implementation study [21,22]. In the Netherlands, we have seen a decrease in the number of new HIV diagnoses since 2010, and since 2018, we have seen a 57% decrease in recent HIV diagnoses. As the effectiveness of PrEP is highly dependent on adherence [1], high discontinuation rates in PrEP programmes could be concerning when individuals are still at risk [23-25]. It therefore is important to provide PrEP users with the tools to correctly stop and (re)start PrEP, so that they can adequately protect themselves against HIV when needed. If discontinuing PrEP places people outside the PrEP programme and limits their access to PrEP when they re-engage in risk activity, their lack of access could stymie HIV prevention, making continued and expanded access to PrEP crucial.

As some HIV infections can remain undiagnosed for many years, temporal trends in the number and proportion of diagnosed recent HIV infections likely provide a more accurate estimation of the effect of PrEP on the HIV epidemic in the Netherlands. While we found a rapid reduction in the number of diagnosed recent HIV infections until 2020, the decrease stagnated thereafter (possibly also due to reduced HIV testing during the COVID-19 pandemic). In the Netherlands, PrEP provision through the National PrEP programme was limited to 8,500 individuals. Consequently, individuals seeking access to PrEP are waitlisted until space becomes available or can access PrEP unsubsidised through other healthcare providers at higher personal costs. We found that, among those newly diagnosed with HIV, at least 16% reported prior PrEP use and unfortunately several acquired HIV while waiting to be enrolled in the Dutch national PrEP programme. Moreover, we showed that a relatively large proportion of individuals were either unaware of PrEP or estimated their own risk of HIV to be low, indicating a need for information campaigns and more adapted counselling. The need for PrEP and proper counselling is further underscored by the decreasing trend of condom use among PrEP users [26-28] and MSM in general [29,30]. From August 2024, PrEP delivery and care has substantially change in the Netherlands. The cost of PrEP tablets is no longer reimbursed and PrEP care visits are reduced to every 6 months. As a result, the capacity of the National PrEP Programme is expanded, but the increased costs and reduced screening may divert participation. Close monitoring of individuals at risk for attrition and contacting individuals who miss screening visits may thus be important to limit unnecessary HIV transmission.

Thirteen individuals harboured HIV strains that showed an M184I/V mutation, associated with resistance against FTC/lamivudine, and two also had a K65R mutation, associated with resistance against TDF. Fortunately, we did not find phylogenetic evidence of the onward transmission of resistance-associated mutations throughout the population of MSM and transgender people newly diagnosed with HIV. Moreover, we found that the prevalence of RT M184V increased after implementing the national PrEP programme. Since mutations at position RT184 are known to rapidly decay due to loss of viral fitness, the actual number of individuals with this mutation is likely higher than we report [31]. In contrast, the increase in RT M184V prevalence may be due to increased HIV testing within the PrEP programme and thus detection of these mutations may also be due to earlier diagnosis [32]. The lower replicative fitness and thus lower transmission risk of M184V might not provide a major concern for individuals using PrEP. However, subsequent selection of tenofovir-resistant strains (K65R/E/N) could lead to PrEP [5-8] and HIV treatment failure. Therefore, monitoring these RAMs remains crucial. While PrEP failure is rare with consistent adherence [1], PrEP users and healthcare providers should be aware of the possibility of PrEP failure.

One strength of our study is the use of a comprehensive database consisting of 98% of all individuals with HIV receiving HIV care in the Netherlands. This provides a unique surveillance tool for not only the HIV epidemic in the Netherlands, but also the effect of biomedical interventions on the HIV epidemic, including surveillance of transmission of clinically-relevant PrEP-associated mutations. Nevertheless, this study is not without limitations. First, information on prior PrEP use was only available for approximately one-third of newly diagnosed MSM and transgender persons, which may have underestimated the number of individuals who used PrEP before HIV diagnosis. Genotypic resistance testing results and polymerase sequences were only available for a subset of individuals who reported prior PrEP use, meaning that for 30 individuals who previously used PrEP, the polymerase sequence was not available for analysis. Second, the National PrEP programme is monitored by the National Institute for Public Health and the Environment, and information regarding PrEP intake, including adherence, cannot be linked to the ATHENA cohort. We thus cannot infer the level of PrEP use before HIV acquisition. Third, as access to PrEP was substantially reduced during the COVID-19 pandemic, this may have affected our results [33]. Lastly, information on PrEP use was self-reported, which can be prone to reporting bias.

## Conclusion

At least 93 MSM and transgender persons used PrEP before HIV acquisition in the Netherlands. We found clinically-relevant PrEP RAMs in 13.8% of these MSM and transgender persons. The overall prevalence of PrEP RAMs in MSM and transgender persons with a recent HIV infection increased from 0.23% to 4.11% after PrEP became available. More widespread access to PrEP, counselling on PrEP use and adherence and retaining people in the PrEP programmes when still at risk for HIV acquisition is crucial to prevent new HIV infections.

## Supplementary Data

249000 Supplementary Material
