# Supplementary material for: Oral HIV pre-exposure prophylaxis use and resistance-associated mutations among men who have sex with men and transgender persons newly diagnosed with HIV in the Netherlands: results from the ATHENA cohort, 2018 to 2022
Source: Euro Surveill. 2024 Sep 19;29(38):2400083. doi: 10.2807/1560-7917.ES.2024.29.38.2400083 (PMC11484289; doi:10.2807/1560-7917.ES.2024.29.38.2400083)

This supplementary material is hosted by *Eurosurveillance* as supporting information alongside the article ‘Oral HIV pre-exposure prophylaxis use and resistance-associated mutations among men who have sex with men and transgender persons newly diagnosed with HIV in the Netherlands: results from the ATHENA cohort, 2018 to 2022’, on behalf of the authors, who remain responsible for the accuracy and appropriateness of the content. The same standards for ethics, copyright, attributions and permissions as for the article apply. Supplements are not edited by *Eurosurveillance* and the journal is not responsible for the maintenance of any links or email addresses provided therein.

Supplementary Figure. Phylogenetic tree of HIV-1 polymerase sequences from 3,054 MSM and transgender persons diagnosed with HIV between 2010 and 2022

The leaf names refer to the subtree assigned as in Table 2. Branches are colored as follows: orange for individuals who have used PrEP; red for individuals who used PrEP and developed related RAMs; blue for individuals with no indication of prior PrEP use, but with PrEP related RAMs; purple for individuals with PrEP related RAMs and singleton in subtrees.

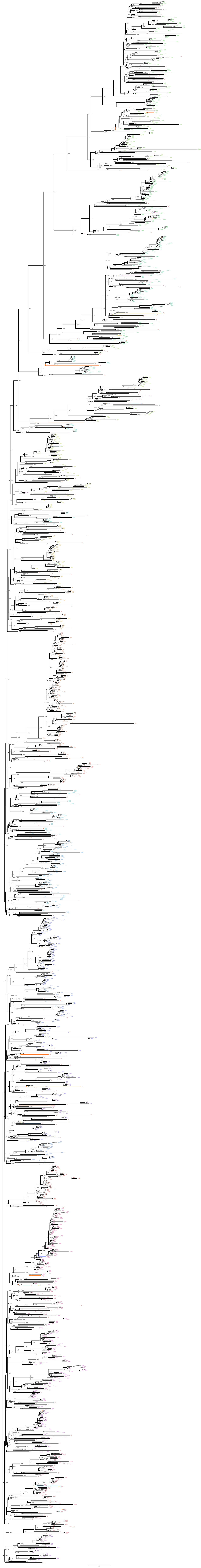
0.03

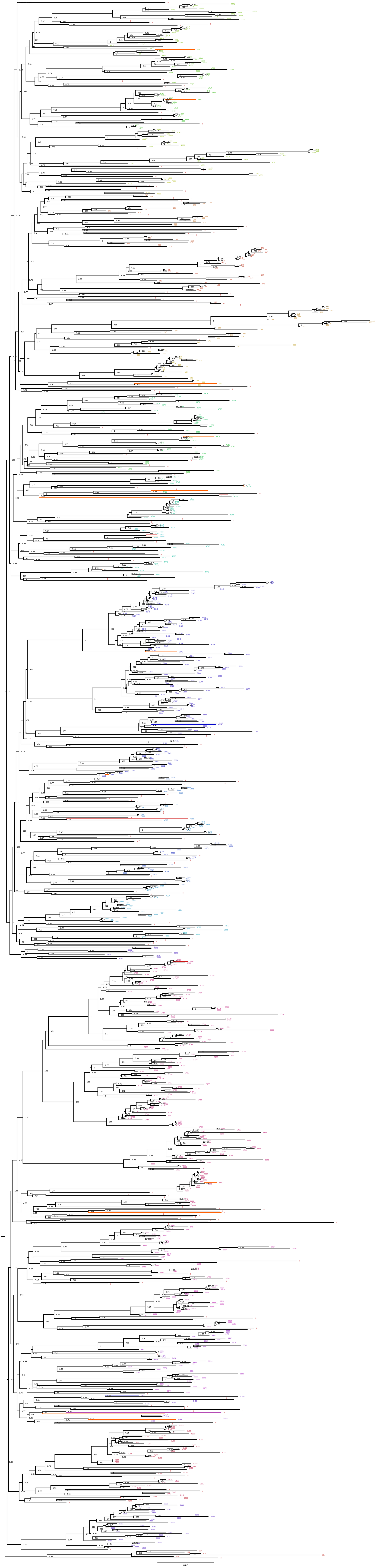

Supplement: Supplementary Material [file 24-00083_JONGEN_Supplement.pdf]
